# Long-term effects of permanent pacemaker therapy on growth in children with complete heart block

**DOI:** 10.3389/fcvm.2026.1856726

**Published:** 2026-08-12

**Authors:** Kutay Sel, Yasemin Nuran Dönmez, Hayrettin Hakan Aykan, Aydın Adıgüzel, İlker Ertuğrul, Tevfik Karagöz

**Affiliations:** Department of Paediatric Cardiology, Hacettepe Universty Ihsan Dogramacı Children’s Hospital, Ankara, Türkiye

**Keywords:** cardiac conduction system, childhood, complete heart block, growth, pacemaker

## Abstract

**Objectives:**

Complete heart block (CHB) is a rare but potentially life-threatening condition in childhood. A permanent pacemaker (PM) can provide a physiologically appropriate heart rate under defined criteria. It is known that normal cardiac function plays an important role in growth, which is the main indicator of health in childhood. While a holistic health approach is increasingly emphasized, there are limited data in the literature evaluating long-term growth in pediatric patients with a PM. This study aimed to assess the impact of PM implantation on growth in children with CHB.

**Methods:**

This study included 25 pediatric patients (11 girls, 14 boys) aged 2–16 years with CHB who underwent PM implantation. Anthropometric measurements, including height, weight, and body mass index (BMI), were recorded pre-implantation and at follow-up, continuing until age 18. *Z* scores adjusted for age and gender were calculated and compared between baseline and final follow-up.

**Results:**

The mean follow-up duration was 7.65 ± 2.79 years. Statistically significant increases were observed in weight and BMI *Z* scores post-implantation (*p* < 0.05). Although height *Z* scores also showed an upward trend, the change did not reach statistical significance.

**Conclusion:**

Permanent PM implantation in children with CHB is associated with significant improvements in weight and BMI over the long term, indicating a positive influence on overall growth. While height gains were less marked, the findings suggest that improved cardiac function post-implantation supports better growth trajectories. These results underscore the importance of timely PM implantation, with favorable growth outcomes observed over long-term follow-up in pediatric patients with CHB.

## 1 Introduction

Complete heart block (CHB) is a condition in which the electrical conduction between the atria and the ventricles of the heart is completely interrupted. Its prevalence in children is low; a population-based study in Japan reported 2 cases per 100.000 asymptomatic school-aged children, with incidence increasing with age (1). Childhood CHB is most often congenital, and permanent pacemaker (PM) implantation remains the only effective treatment (2).

Despite widespread PM use in pediatric CHB, data on its long-term effects on growth is limited. This study evaluates growth parameters of children with CHB before PM implantation and at follow-up, based on the hypothesis that PM therapy improves growth by optimizing cardiac output.

## 2 Materials and methods

The study was limited to pediatric patients with isolated, complete, permanent blocks. Complete heart block was defined as electrocardiographic evidence of atrioventricular dissociation, with atrial rate exceeding the ventricular rate. The decision to implant a PM was made according to the criteria specified in the guidelines (3). The medical records of children who underwent permanent PM implantation for CHB between the ages of 2 and 16 years were systematically reviewed. Patients who underwent PM implantation before the age of 2 years or after the age of 16 years were excluded to allow a sufficiently long and comparable follow-up period for growth evaluation. To minimize the potential influence of pacing-induced ventricular dysfunction on growth outcomes, only patients with preserved left ventricular systolic function (ejection fraction >55%) throughout follow-up were included. Patients who developed different systemic diseases during follow-up, died due to various reasons, and dropped out of follow-up were excluded. Patients with a history of premature birth, syndromic patients, and patients with structural heart disease were also excluded. Patients’ complaints at presentation, age at presentation, 24-h rhythm holter findings before PM indication, duration of PM-free follow-up, and age at the time of PM implantation were recorded. Anthropometric data (height in cm, weight in kg) were collected at baseline (pre-implantation) and at the final visit (up to age 18). Body Mass Index (BMI) was calculated using the standard formula (kg/m^2^). All growth variables were converted into age-and gender-adjusted *Z*-scores (standard deviation scores-SDS) using the “Child Metrics program” according to the data of the Turkish child population in the study of Neyzi et al. (4). The corresponding growth percentiles were also calculated using the same reference standards for clinical interpretation; however, all statistical analyses were based on *Z*-scores. The nutritional status of patients was classified using age- and sex-adjusted BMI *Z* scores based on the Neyzi reference curves. Patients were categorized as underweight if BMI *Z* score was <−2 SDS, normal weight if between −2 and +1 SDS, overweight if >+1 to ≤+2 SDS, and obese if >+2 SDS. The baseline values of the patients were compared with the final values at the end of the follow-up period. Informed consent was obtained from the parent of all the subjects involved in this study**.** The study was conducted in accordance with the Declaration of Helsinki, and approved by the Ethics Committee of Hacettepe University (2020-1251).

### 2.1 Statistics

All statistical analyses were performed using SPSS version 17.0. These included descriptive statistics and paired comparisons. Values for numerical variables were given as mean ± standard deviation or median (min–max), depending on the normality of the distribution. Normality of distribution of numerical variables was assessed by Shapiro–Wilk tests. For repeated measurements showing a normal distribution, the paired samples *t*-test was used to compare anthropometric values at the time of PM insertion with final values at follow-up. An overall *p*-value of less than 0.05 was considered statistically significant.

## 3 Results

A total of 25 patients (11 females, 14 males) diagnosed with CHB between January 2000 and January 2015 were included in the study. The most common diagnosis was recognition during routine examination (*n* = 8, 32%). Other complaints leading to the diagnosis were murmur (*n* = 6, 24%), chest pain (*n* = 3, 12%), dizziness, fatigue, seizures and syncope, respectively.

The median age of the patients at the time of diagnosis was 5 years (mean: 5.89 ± 3.60 years) and the mean age at the time of PM implantation was 7.39 ± 3.64 years. At diagnosis, 14 patients already met criteria for PM implantation, whereas 11 were followed for a median of 24 months (range: 6–135 months) before implantation. The mean heart rate on the 24-h rhythm holter was 50 ± 4/min before the PM decision. At the time of the initial PM implantation, 9 patients received epicardial pacing systems, while the remaining 16 patients underwent endocardial PM implantation. Consistent with the study inclusion criteria, all patients maintained preserved left ventricular systolic function throughout follow-up, with no echocardiographic evidence of clinically significant pacing-induced ventricular dysfunction. The mean follow-up period until the age of 18 years was 7.65 ± 2.79 years (median 7 years, range: 2–15 years). The general characteristics of our patients are given in Table 1.

**Table 1:** Clinical characteristics of children with complete heart block included in the study.

Patients, *n* = 25 (11 female)	Mean ± SD (Min–Max)
Age at diagnosis (years)	5.89 ± 3.60 (1–16)
Pre-PM follow-up duration in 11 patients (months)	40.9 ± 41.53 (6–135)
Mean heart rate before PM (beats/min)	50 ± 4 beats/min (41–58)
Age at the time of PM insertion (years)	7.39 ± 3.64 (2–16)
Mean follow-up duration to 18 years of age (years)	7.65 ± 2.79 (2–15)

PM, pacemaker; SD, standard deviation.

Values are presented as mean ± SD.

**Figure 1:**
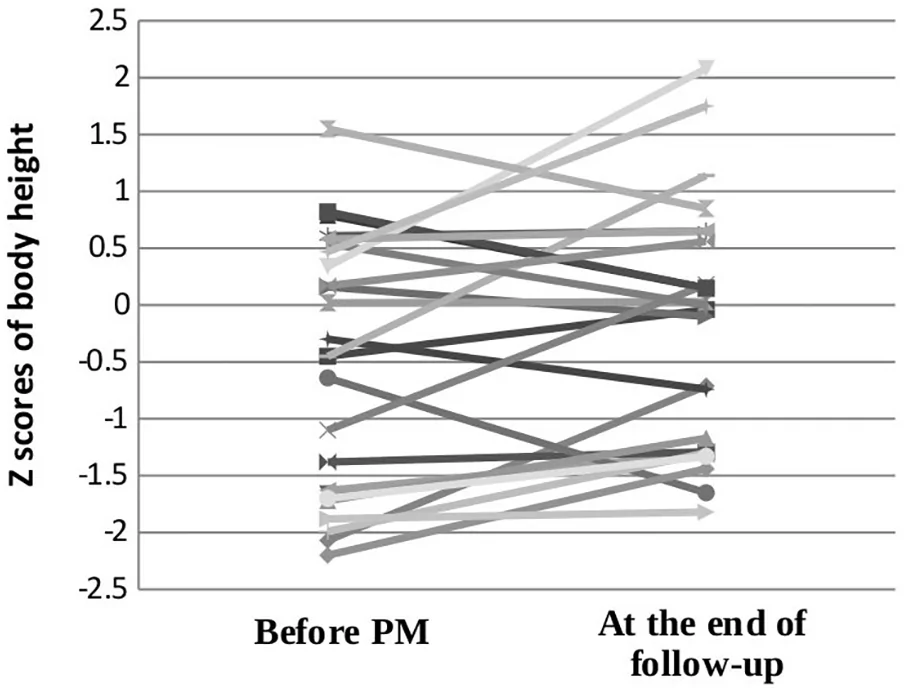
Individual changes in body height *Z*-scores from baseline (before PM implantation) to the end of follow-up. Each line represents one patient. PM, permanent pacemaker.

**Figure 2:**
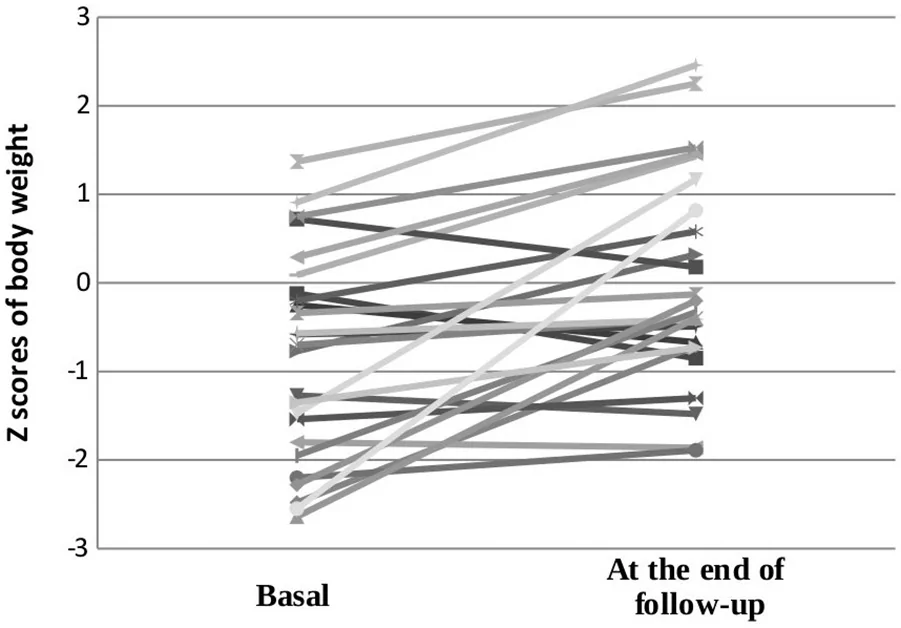
Individual changes in body weight *Z*-scores from baseline (before PM implantation) to the end of follow-up. Each line represents one patient. PM, permanent pacemaker.

We compared the height and weight values of these 25 patients at the time of PM implantation and at the end of follow-up. The mean baseline and end-of-follow-up weight *Z* scores were −0.83 ± 1.17 and 0.01 ± 1.19, respectively. The mean baseline and end of follow-up body height *Z* scores were −0.52 ± 1.11 and −0.24 ± 1.08, respectively. When the baseline and end of follow-up values of the patients were compared, the change in weight was significantly different (*p* < 0.05). The change in height was not statistically significant (*p* = 0.071). At the end of the follow-up, the weight *Z* score change was calculated as 0.89 ± 0.99 and the height *Z* score change as 0.32 ± 0.73. For ease of clinical interpretation, the corresponding mean weight percentile increased from 30.0 to 47.5, while the mean height percentile increased from 37.7 to 42.8. Table 2 summarizes the anthropometric changes from baseline to final follow-up. Additionally, changes in the patients’ height and weight *Z* scores are shown in Figures 1, 2. At the end of follow-up, 21 of 25 patients (84%) demonstrated an increase in weight *Z*-score, while 18 of 25 (72%) showed improvement in height *Z*-score. It was observed that 3 patients with baseline weight *Z* score less than −2 showed catch-up growth. Two patients with both baseline weight and height *Z* scores less than −2 were seen to catch up in both parameters at the end of the follow-up.

**Table 2:** Anthropometric values of patients before and after pacemaker.

Parameter	Baseline *Z*-score (mean ± SD)	End-of-follow up *Z*-score (mean ± SD)	*p* value	Mean percentile change
Height	−0.52 ± 1.11	−0.24 ± 1.08	*p* = 0.071	37.7→42.8
Weight	−0.83 ± 1.17	0.01 ± 1.19	***p*** **<** **0.05**	30.0→47.5
BMI	−0.86 ± 1.30	0.11 ± 1.00	***p*** **<** **0.05**	32.0→52.9

SD, standard deviation; BMI, body mass index.

Values are presented as mean ± SD. *Z*-scores and percentiles were calculated according to age- and sex-specific reference standards. Percentiles are provided for clinical interpretation only; statistical analyses were performed using *Z*-scores. Statistically significant *p*-values ​​are shown in bold.

**Figure 3:**
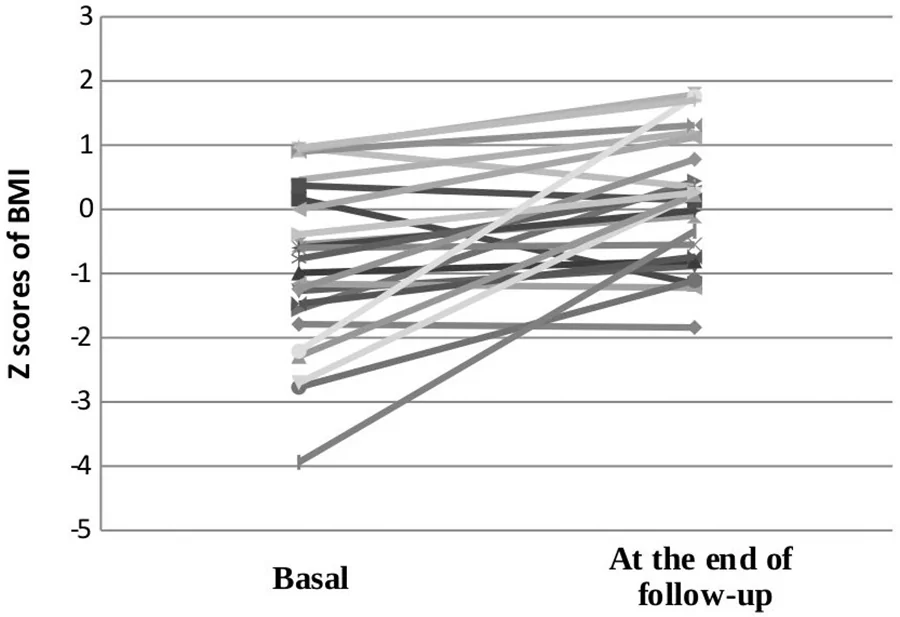
Individual changes in BMI *Z*-scores from baseline (before PM implantation) to the end of follow-up. Each line represents one patient. BMI, body mass index; PM, permanent pacemaker.

The mean baseline and end-of-follow-up BMI *Z* scores were −0.86 ± 1.30 and 0.11 ± 1.00, respectively. The mean change in BMI of these patients was 0.93 ± 1.25. The change in BMI values was statistically significant when baseline and follow-up values were compared (*p* < 0.05). The corresponding mean BMI percentile increased from 32.0 to 52.9. Figure 3 shows the *Z*-score changes in BMI. Based on age- and sex-adjusted BMI *Z*-score classification, 5 patients (20%) were underweight before PM implantation, whereas no patients remained underweight at the final follow-up. No patients were overweight or obese before PM implantation; however, 6 patients (24%) were classified as overweight at the final follow-up, while none met the criteria for obesity.

## 4 Discussion

The mean heart rate decreases with age from the neonatal period to adulthood (5). Similarly, basal metabolism is faster in children as a growing organism and decreases with age (6). Adequate cardiac output must be provided to meet the needs of these growing tissues. In young children and especially neonates, the myocardium is less compliant than in adults and therefore children are less able to increase stroke volume than adults. The ability of cardiac cells to regulate stroke volume increases with age. In other words, heart rate, one of the two determinants of cardiac output, is more effective in childhood (7, 8).

Complete heart block is a rare but serious condition requiring close follow-up, and permanent PM implantation remains the only effective treatment. The major challenge in childhood is that most recommendations are based on levels of evidence B and C, making it difficult to offer strong guidance. Indications for pacing in children differ from adults due to age-specific physiology and the variable clinical expression of conduction disorders. In pediatric patients, permanent pacing is generally recommended in cases of symptomatic bradycardia, advanced atrioventricular block (particularly postoperative or congenital CHB with low ventricular rates), and conditions in which bradyarrhythmia leads to impaired ventricular function An important concern is that the expected lifespan with a PM is considerably longer in children. In the 2021 guideline, unlike previous recommendations, the heart-rate thresholds were lowered specifically to prevent unnecessary early PM implantation in children with CHB (9). The importance of delaying permanent PM implantation as much as possible was also emphasized in order to reduce complication risks. While guidelines are crucial for making treatment decisions, it should also be remembered that each patient has conditions that need to be evaluated individually. Our patients in the study were evaluated in accordance with the guideline recommendations that were in effect at the time the decision for PM was made.

In our study, CHB patients whose cardiac output restored to age-appropriate physiological levels after PM implantation showed increases in both height and weight, with the weight gain reaching statistical significance. These results suggest favorable growth outcomes during follow-up after PM implantation.

In a study of newborns exposed to anti-Ro antibodies during the fetal period, Skog et al. reported that infants with second- and third-degree heart block had significantly lower gestational age-adjusted body weight, body length, and head circumference compared with those with first-degree block or no block. They also observed that these patients did not achieve catch-up growth during infancy (10). In a subsequent study of Skog et al. with extended follow-up, the authors noted that these children eventually reached normal growth parameters and caught up with their healthy siblings (11). They further reported that PM treatment was not associated with growth recovery; however, only changes in weight were evaluated, and the maximum follow-up period after PM implantation was limited to one year. In our study, which had a follow-up period of nearly eight years, both weight and height parameters were evaluated. A similar aspect to Skog's is that patients are more affected in terms of weight. They reported that their patients had over 2SD weight at rates ranging from 12% to 21% depending on their age group (11). Steele et al. drew attention to the increased risk of obesity in pediatric patients with electrophysiologic disorders (12). In our cohort, no patients were overweight before PM implantation, whereas six patients were classified as overweight at the end of follow-up based on age- and sex-adjusted BMI *Z*-score criteria. Although physical activity was not assessed in our study, the observed increase in BMI may also reflect reduced habitual physical activity related to parental overprotection, as suggested in previous studies. In addition to behavioral factors, physiological changes after PM implantation may also have contributed to this finding. Restoration of an age-appropriate heart rate improves cardiac output and reduces the chronic hemodynamic burden associated with severe bradycardia, which may favor weight gain during long-term follow-up. These findings emphasize the importance of regular nutritional monitoring and lifestyle counseling during long-term follow-up.

## 5 Conclusions

Early recognition of CHB in children is crucial, even in asymptomatic cases. Permanent PM implantation is safe and the only treatment option for these patients. In our study, favorable growth outcomes were observed in CHB patients who underwent PM implantation. While growth outcomes are not definitive indications for PM implantation, they may play a supporting role in clinical decision-making and counseling families about long-term expectations.

## 6 Limitations

We formed a homogeneous group for a rare disease, therefore the number of patients was limited. One limitation of our study is that we do not have clear information about the age of onset of CHB in our patients. Congenital forms, the most common cause, can remain asymptomatic until later in life (13, 14).

The retrospective pre-post design without a disease-matched control group precludes definitive causal attribution of the observed changes in growth outcomes to PM implantation. In addition, establishing a long-term non-paced control group in children with CHB is ethically and practically challenging, as permanent PM implantation is the standard treatment for clinically indicated patients.

## Data Availability

The raw data supporting the conclusions of this article will be made available by the authors, without undue reservation.
